# The effect of different volumes and temperatures of saline on the bladder pressure measurement in critically ill patients

**DOI:** 10.1186/cc6080

**Published:** 2007-07-26

**Authors:** Davide Chiumello, Federica Tallarini, Monica Chierichetti, Federico Polli, Gianluigi Li Bassi, Giuliana Motta, Serena Azzari, Cristian Carsenzola, Luciano Gattinoni

**Affiliations:** 1 Dipartimento di Anestesia e Rianimazione, Fondazione IRCCS – 'Ospedale Maggiore Policlinico, Mangiagalli, Regina Elena', Via F Sforza 35, 20122 Milan, Italy; 2 Istituto di Anestesia e Rianimazione Università degli Studi di Milano, 'Ospedale Maggiore Policlinico, Mangiagalli, Regina Elena', Via F Sforza 35, 20122 Milan, Italy

## Abstract

**Introduction:**

Intra-abdominal hypertension is common in critically ill patients and is associated with increased severity of organ failure and mortality. The techniques most commonly used to estimate intra-abdominal pressure are measurements of bladder and gastric pressures. The bladder technique requires that the bladder be infused with a certain amount of saline, to ensure that there is a conductive fluid column between the bladder and the transducer. The aim of this study was to evaluate the effect of different volumes and temperatures of infused saline on bladder pressure measurements in comparison with gastric pressure.

**Methods:**

Thirteen mechanically ventilated critically ill patients (11 male; body mass index 25.5 ± 4.6 kg/m^2^; arterial oxygen tension/fractional inspired oxygen ratio 225 ± 48 mmHg) were enrolled. Bladder pressure was measured using volumes of saline from 50 to 200 ml at body temperature (35 to 37°C) and room temperature (18 to 20°C).

**Results:**

Bladder pressure was no different between 50 ml and 100 ml saline (9.5 ± 3.7 mmHg and 13.7 ± 5.6 mmHg), but it significantly increased with 150 and 200 ml (21.1 ± 10.4 mmHg and 27.1 ± 15.5 mmHg). Infusion of saline at room temperature caused a significantly greater bladder pressure compared with saline at body temperature. The lowest difference between bladder and gastric pressure was obtained with a volume of 50 ml.

**Conclusion:**

The bladder acts as a passive structure, transmitting intra-abdominal pressure only with saline volumes between 50 ml and 100 ml. Infusion of a saline at room temperature caused a higher bladder pressure, probably because of contraction of the detrusor bladder muscle.

## Introduction

Intra-abdominal pressure (IAP) is the pressure generated inside the abdominal cavity and depends on the degree of flexibility of the diaphragm and abdominal wall, and on the density of its contents [1]. Intra-abdominal hypertension (IAH), defined as an abnormal increase in IAP, can be common in critically ill patients, being present in 18% to 81% of the patients depending on the cut-off level used [2-8].

Several clinical conditions such as accumulation of blood, ascites, retroperitoneal haematoma, bowel oedema, necrotizing pancreatitis, massive fluid resuscitation, packing after control laparotomy and closure of a swollen noncompliant abdominal wall may induce IAH [3,9]. IAH has adverse effects on several organs, causing reductions in cardiac output [10], deterioration in gas exchange [11-13] and decreases in splachnic-renal perfusion [14-16]. In surgical [17], trauma [2] and medical [6] critically patients, the IAH was an independent predictor factor of hospital mortality. Although surgical decompression remains the only definitive therapy in the case of substantial IAH, and the IAP is lower after decompression, mortality remains considerable [18,19].

Because the abdomen and its contents can be considered to be relatively noncompressive and fluid in character, behaving in accordance with Pascal's law, the IAP measured at one point is assumed to reflect the IAP throughout the abdomen [4]. A variety of methods for measuring IAP have been proposed, which are either indirect (by transduction of bladder, gastric, or uterine pressure using a ballon catheter) or direct (using a intraperitoneal catheter) [1,20]. However, among the different methods, the intra-bladder pressure (IBP) technique is the most commonly used because of its simplicity and low cost [4,21].

The bladder technique, originally described by Kron and coworkers [14], assumes that the bladder behaves like a passive pressure membrane transducer when it is infused with a small amount of saline [14]. However, various saline volumes for bladder priming, 50 ml up to 250 ml, have been used to estimate IBP [10,14,21-23]. Previous studies demonstrated that a small volume of saline (10 to 25 ml) is required to prime the bladder in order to avoid overestimating the IBP [22,24,25]. The International Abdominal Compartment Syndrome Consensus Conference [1] suggested that a maximal instillation volume of 25 ml of saline should be used. In addition the bladder – being a muscular organ – may change its elasticity in response to various external stimuli, such as an infusion of warm saline [26]. Thus the bladder may not always behave like a passive elastic structure, leading to inaccurate estimation of IAP.

The aim of this study was to evaluate IAP estimated by bladder pressure, measured with the bladder infused with different volumes of saline at room and body temperatures, in comparison with intra-gastric pressure (IGP).

## Materials and methods

### Study population

Thirteen sedated, mechanically ventilated patients admitted to the intensive care unit of Ospedale Policlinico were enrolled. Exclusion criteria were contraindications to bladder pressure measurement (a recent history of bladder surgery, haematuria, trauma, or neurogenic bladder).

The study was approved by the institutional review board of our hospital, and informed consent was obtained in accordance with Italian national regulations.

### Study protocol

The IBP was measured using a revision of the Cheatham's original technique [21] with disposable pressure transducer (Edward Lifesciences, Irvine, CA, USA). A 18-gauge needle was inserted into the culture aspiration port of the Foley's catheter and connected with a sterile tube to the pressure transducer using two three-way stopcocks. A standard infusion bag of normal saline was attached to one stopcock and a 60 ml syringe was connected to the second stopcock. Before taking any measurements, the system was flushed with sterile saline and the pubic symphysis was always used as zero reference point with the subject in the complete supine position.

The IBP was measured at different volumes of saline infusion (50, 100, 150 and 200 ml, with steps of 50 ml) at room temperature (18 to 20°C). The sequence of measurements was then repeated using saline infusion warmed to body temperature (35 to 37°C). At each volume of saline, the IBP was recorded 5 to 10 s after the termination of saline infusion (early recording) and 5 min later (late recording) by keeping the bladder catheter closed. After each measurement the bladder was emptied.

Each patient was studied at an external positive end-expiratory pressure of 10 cmH_2_O, with the other ventilatory parameters (previously selected by the attending physician) unchanged during the study. Thus, each patient underwent two randomized series of measurements.

The IGP was measured using a radio-opaque balloon (SmartCath; Bicore, Irvine, CA, USA) connected to a pressure transducer (Bentley Trantec; Bentley Laboratories, Irvine, CA, USA) [27]. For measurement purposes, the gastric balloon was inflated with 1.0 ml air.

The IBP and IGP were measured at end-expiration and the signals were recorded on a personal computer for subsequent analysis (Colligo; Elekton, Milan, Italy).

The level of sedation before the study was evaluated using the Ramsay scale [28]. The Simplified Acute Physiology Score II was used to assess the severity of systemic illness at study entry [29], whereas the Sepsis Related Organ Failure Assessment score was computed on the day of the study by considering the worst value for each organ system (respiratory, cardiovascular, renal, coagulation, liver and neurological) [30].

### Statistical analysis

The effects of volume, temperature of saline infused and time of recording were analyzed by two-way repeated measures analysis of variance, followed by Student/Newman Keuls test for multiple comparison (SigmaStat 2.03; SPSS Inc., Chicago, IL, USA) [31]. *P *< 0.05 was considered statistically significant.

The mean bias (bladder minus gastric pressure), precision (standard deviation of the bias) and limits of agreement were calculated using the Bland-Altman analysis [32]. The percentage error was calculated in accordance with the method proposed by Crichley and coworkers [33].

All data are expressed as mean ± standard deviation.

## Results

The main clinical characteristics are reported in Table 1. The patients were studied after a mean of 6 ± 3.8 days from intensive care admission.

**Table 1:** Patient's characteristics

Patient	Age (years)	BMI (kg/m^2^)	Sex	SAPS II score	SOFA	PEEP (cmH_2_O)	PaO_2_/FiO_2 _(mmHg)	MAP (mmHg)	Hourly urine output (ml/hour)	Ramsay score	Diagnosis	Outcome
1	72	30.9	M	35	4	10	218	100	80	7	Sepsis	D
2	83	26.3	M	48	8	10	285	67	100	5	Sepsis	S
3	70	26.2	M	40	8	2	228	107	60	4	Sepsis	S
4	72	26.0	M	32	8	6	208	100	60	5	Sepsis	S
5	65	34.6	F	47	5	10	173	100	100	5	Sepsis	S
6	55	20.2	F	36	7	13	230	68	100	6	Sepsis	S
7	43	24.9	M	26	15	13	211	84	140	5	Sepsis	S
8	87	27.8	M	41	3	17	280	100	80	7	Sepsis	S
9	72	26.3	M	35	5	15	240	100	100	7	ALI post surgery	S
10	77	16.4	M	43	12	15	170	100	80	7	ARDS	D
11	56	19.6	M	27	2	8	288	100	200	6	Sepsis	D
12	79	24.8	M	53	13	5	133	87	50	7	Sepsis	D
13	74	27.8	M	46	9	5	195	80	110	7	Sepsis	S
Total or mean ± SD	68 ± 13	25.5 ± 4.6	11 M/2 F	7.7 ± 3.8	9.5 ± 4.6	9.5 ± 4.6	225 ± 48	92 ± 13	96 ± 39	6 ± 1		4 D/9 S

The Simplified Acute Physiology Score (SAPS) II was used to assess the severity of systemic illness at study entry. The Sepsis-Related Organ Failure Assessment (SOFA) was used to assess the organ failure at the day of the study. ALI, acute lung injury; ARDS, acute respiratory distress syndrome; BMI, body mass index; D, dead; F, female; M, male; MAP, mean arterial pressure; PEEP, positive end-expiratory pressure; S, survived; SD, standard deviation.

**Figure 1:**
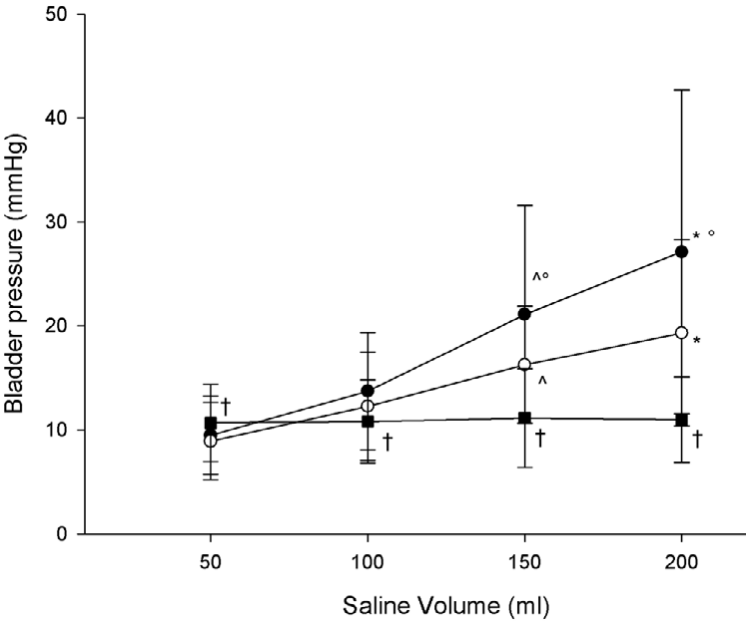
IBPs measured at different volumes of saline and IGP: early versus late. Shown are the intra-bladder pressures (IBPs) measured at different volumes of saline (black circle indicates early recording, and white circle indicates late recording) and intra-gastric pressure (IGP; black square) at 10 cmH_2_O of positive end-expiratory pressure. ^*P *< 0.05 versus 50 and 100 ml saline; *P < 0.05 versus 50, 100, and 150 ml saline; °*P *< 0.05 versus late recording; ^†^*P *< 0.05 versus intra-bladder pressure.

The IBP was no different with 50 and 100 ml volumes of saline (9.5 ± 3.7 mmHg and 13.7 ± 5.6 mmHg; *P *= 0.071), but it was significantly higher with 150 and 200 ml saline (21.1 ± 10.4 and 27.1 ± 15.5 mmHg; *P *< 0.001; Figure 1). Considering the IBP measured with 50 ml of saline infused as reference, we computed the agreements with the IBP measured with 100, 150 and 200 ml of saline (Table 2).

**Table 2:** Agreement analysis between bladder pressure and bladder pressure

Volumes of saline (ml)	Mean (mmHg)	Bias (mmHg)	Precision (mmHg)	Lower limits of agreement (mmHg)	Upper limits of agreement (mmHg)	Percentage error
100	13.7	4.2	2.9	-1.4 (-4.5 to +1.6)	9.9 (6.9 to 13.0)	± 41%
150	21.1	11.2	9.8	-8.0 (-18.7 to +2.8)	30.4 (19.6 to 41.2)	± 91%
200	27.1	17.6	14.7	-11.1 (-26.4 to +4.3)	46.4 (31.0 to 61.7)	± 106%

Shown is an agreement analysis between bladder pressure measured with 50 ml saline (as reference) and that bladder pressure measured with 100, 150 and 200 ml saline. The bias, precision, limits of agreement and percentage error were computed considering intra-bladder pressure (IBP) at 50 ml versus IBP at 100, 150 and 200 ml.

Four patients (30.7% of the population) were classified as having IAH (IAP >12 mmHg) when 50 ml saline was used. This increased to eight patients (61.5% of the population) when 100 ml saline was used.

**Figure 2:**
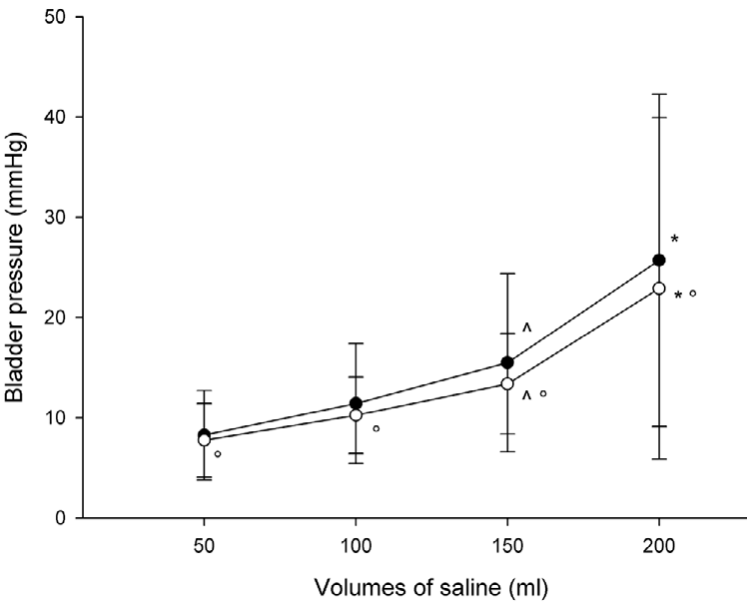
IBPs measures at different volumes of saline: saline at room temperature versus body temperature. The intra-bladder pressure (IBP) measured at the different volumes of saline (black circle indicates saline at room temperature, and white circle indicates saline at body temperature). ^*P *< 0.05 versus 50 and 100 ml saline; *P < 0.05 versus saline at room temperature.

The IBP was significantly lower 5 min after saline infusion (late recording) than just after the saline infusion (early recording), but only with the bladder infused with 200 and 150 ml of saline (21.1 ± 10.4 versus 16.2 ± 5.6 mmHg, and 27.1 ± 15.5 versus 19.3 ± 8.9 mmHg; *P *< 0.005; Figure 1). At each volume infused, the infusion of saline at body temperature resulted in a significantly lower IBP than did infusion of saline at room temperature (8.2 ± 4.4 versus 7.7 ± 3.7 mmHg with 50 ml saline, 11.4 ± 5.9 versus 10.2 ± 3.8 mmHg with 100 ml saline, 15.4 ± 8.8 versus 13.3 ± 5.0 mmHg with 150 ml saline, and 25.7 ± 16.5 versus 22.8 ± 17.0 mmHg with 200 ml saline; *P *< 0.001; Figure 2). The differences between the paired measurements of IGP and IBP (bias) are given in Table 3. The lowest bias was found for a 50 ml volume of saline, whereas the bias increased with increasing the volume of saline infused in the bladder.

**Table 3:** Agreement analysis between bladder and gastric pressure

Volumes of saline (ml)	Mean (mmHg)	Bias (mmHg)	Precision (mmHg)	Lower limits of agreement (mmHg)	Upper limits of agreement (mmHg)	Percentage error
50	9.5	1.2	4.3	-7.2 (-11.7 to -2.7)	9.6 (5.1 to 14.1)	± 89%
100	13.7	-2.9	6.3	-15.3 (-21.9 to -8.7)	9.5 (2.8 to 16.1)	± 90%
150	21.1	-9.9	13.0	-35.4 (-49.8 to -21.1)	15.6 (1.3 to 30.0)	± 121%
200	27.1	-16.2	17.9	-51.2 (-69.9 to -32.5)	18.8 (0.1 to 37.5)	± 129%

Shown is an agreement analysis between bladder pressure (measured at different volumes of saline) and gastric pressure. The bias, precision, limits of agreement and percentage error were computed considering intra-bladder pressure versus intra-gastric pressure at each volume of saline infused.

## Discussion

The major findings of this study were as follows. First, increasing the volume of saline infused led to higher IBP. Second, the IBP was significantly lower when measured after 5 min compared with when it was measured just after the termination of the volume infusion, but only with 150 and 200 ml saline. Third, the IBP was significantly lower when measured with infusion of saline at body temperature compared with saline at room temperature. Finally, the lowest bias between the IBP and IGP was obtained with the bladder infused with 50 ml saline.

An increase in IAP is associated with various organ dysfunctions (local and systemic), which in turn are associated with significantly increased in morbidity and mortality [1]. Despite these potential adverse clinical consequences, however, IAP is commonly measured only when there is some clinical suspicion; furthermore, there is currently no general consensus on how frequently it should be measured [34]. Sugrue and coworkers [35] found that clinical examination alone was not accurate in estimating IAP, finding that the likelihood of physicians correctly identifying IAH was lower than 50%. Thus, accurate estimation of IAH is fundamental to appropriate and timely patient management [36].

The most widely used technique to measure the IAP is the bladder pressure technique, as proposed by Kron and coworkers [14]. In that study the authors found that the IBP measured using saline volumes between 50 and 100 ml through a Foley catheter correlated well with pressures measured using a peritoneal dialysis catheter during several infusions of peritoneal dialysis solution. Iberti and colleagues [10], in a canine model of increased IAP, estimated bladder pressure with the bladder empty; they demonstrated that the IBP accurately reflected the IAP. Fusco and coworkers [22], using a human model in which IAP ranged between 0 and 25 mmHg during laparoscopic surgery, found that the bladder emptied (with a volume of 0 ml) yielded the most accurate estimation of IAP. However, at an IAP of 25 mmHg the bladder volume exhibiting the lowest bias was 50 ml.

In the present study, although we did not find any statistically significant difference (there was only a trend) in IBP measured using saline volumes of 50 and 100 ml, this difference could lead to a patient being incorrectly identified as having IAH if a 100 ml rather than a 50 ml of volume were used. Similarly, De Waele and colleagues [24] demonstrated that 12 patients were categorized as suffering from IAH when a volume of 10 ml was used, increasing to 15 and 17 patients, respectively, when 50 and 100 ml volumes were used. Previous studies conducted in adult patients [22,24,25] found that the increase in IBP was statistically significant with a small instillation volume, and two studies conducted in children and infants [37,38] found that the IAP is most accurately measured by instilling into the bladder 1 ml saline per kilogram of body weight. Thus, it has been proposed that the appropriate amount of volume is that required to create a fluid column without interposed air [39].

Although these findings clearly indicate that the IBP can overestimate IAP when large volumes of saline are infused, the possible mechanisms involved are still not clearly understood. The bladder is a muscular membranous organ that is composed of four layers, namely mucous, adventitia, serosa and muscularis, and its elasticity decreases in response to a direct mechanical increase in stress and strain on its structure (when a large amount of saline is infused). In addition, the elasticity of bladder can also be reduced by contraction of the detrusor muscle, mediated by sensory receptors located in the bladder wall, after a rapid infusion of saline or other fluid that is not at body temperature [26].

A recording of bladder pressure 5 min after termination of the infusion yielded a significantly lower IBP only with a volume of saline up to 150 ml; this suggests that the bladder takes longer to reach a stable condition only when it is infused with large volumes. However, this is not relevant in current clinical practice, because the IAP is usually measured with volumes of saline lower than 150 ml.

We found that infusion of saline at body temperature, at each volume infused, also resulted in a significantly lower IBP compared with infusion of saline at room temperature. Rapid infusion of saline at a temperature lower than body temperature may activate contraction of the detrusor muscle (as mentioned above) by a reflex loop through nociceptors with C afferent fibres located in the bladder wall [26], causing a falsely elevated IAP recording.

Another possible cause of reduced elasticity of the bladder might be continued urine drainage through the catheter [40]. In critically ill patients, De Waele and coworkers [24] observed a direct relationship between the duration of catheterization and the difference in bladder pressure measured using volumes of saline of 10 and 100 ml. This suggests that the bladder should be filled only minimally if an accurate measurement of IAP is to be obtained, especially in patients with prolonged catheterization.

In cases of bladder trauma, pelvic fractures or haematoma, or neurogenic bladder, in which the bladder pressure technique cannot be applied, the IGP technique is recommended [1]. Compared with the IBP technique, IGP measurements do not interfere with urine output and avoid risk for infection [22]. In critically ill patients and in patients undergoing laparoscopic cholecystectomy with the abdominal cavity inflated at a pressure of 20 mmHg, a clinically acceptable agreement between IGP and IBP was observed [41,42]. Unexpectedly, we found much greater limits of agreement, probably because of the presence of gastric motor activity, which falsely increases 'true' estimation of IAP.

## Conclusion

In clinical practice the IAP should be estimated using the IBP technique, infusing the bladder with only a small amount of volume of saline at body temperature to avoid overestimating the IAP. If this is not feasible, then the IGP should be measured.

## Key messages

• In clinical practice, IAP should be estimated using the IBP technique with the bladder infused with only a small volume of saline.

• The saline infused should be at body temperature to avoid overestimating the IAP.

• It is recommended that sufficient equilibration time be allowed before the IAP is measured.

• IGP correlates with IBP only at low volumes of saline.

## Abbreviations

IAH = intra-abdominal hypertension; IAP = intra-abdominal pressure; IBP = intra-bladder pressure; IGP = intra-gastric pressure.

## Competing interests

The authors declare that they have no competing interests.

## Authors' contributions

DC conceived of the study, participated in its design and coordination, performed the measurements and wrote a first draft of the manuscript. FT participated in the study design and coordination, performed the measurements and to helped draft the manuscript. MC participated in the study design and coordination, and performed the measurements. FP performed the statistical analysis and helped to draft the manuscript. GLB participated in the study design and coordination, and performed the measurements. GM participated in the study design and coordination, and performed the measurements. SA participated in the study design and coordination, and performed the measurements. CC participated in the study design and coordination, and performed the measurements. LG conceived the study, participated in its design and coordination, coordinated the final analysis of collected data and revised the manuscript, writing its final version.
